# The ETS-Domain Transcription Factor Elk-1 Regulates COX-2 Gene Expression and Inhibits Glucose-Stimulated Insulin Secretion in the Pancreatic ****β****-Cell Line INS-1

**DOI:** 10.1155/2013/843462

**Published:** 2013-06-02

**Authors:** Xiong-Fei Zhang, Yi Zhu, Wen-Biao Liang, Jing-Jing Zhang

**Affiliations:** ^1^Department of Biochemistry, Wenzhou Medical College, Wenzhou 325035, China; ^2^Department of General Surgery, The First Affiliated Hospital of Nanjing Medical University, 300 Guangzhou Road, Nanjing 210029, China; ^3^Jiangsu Province Academy of Clinical Medicine, Institute of Tumor Biology, 300 Guangzhou Road, Nanjing 210029, China; ^4^Transfusion Laboratory, Jiangsu Province Blood Center, Nanjing 210029, China

## Abstract

Cyclooxygenase-2 (COX-2) expression is associated with many aspects of physiological and pathological conditions, including pancreatic **β**-cell dysfunction. Prostaglandin E2 (PGE2) production, as a consequence of COX-2 gene induction, has been reported to impair **β**-cell function. The molecular mechanisms involved in the regulation of COX-2 gene expression are not fully understood. We previously demonstrated that transcription factor Elk-1 significantly upregulated COX-2 gene promoter activity. In this report, we used pancreatic **β**-cell line (INS-1) to explore the relationships between Elk-1 and COX-2. We first investigated the effects of Elk-1 on COX-2 transcriptional regulation and expression in INS-1 cells. We thus undertook to study the binding of Elk-1 to its putative binding sites in the COX-2 promoter. We also analysed glucose-stimulated insulin secretion (GSIS) in INS-1 cells that overexpressed Elk-1. Our results demonstrate that Elk-1 efficiently upregulates COX-2 expression at least partly through directly binding to the −82/−69 region of COX-2 promoter. Overexpression of Elk-1 inhibits GSIS in INS-1 cells. These findings will be helpful for better understanding the transcriptional regulation of COX-2 in pancreatic **β**-cell. Moreover, Elk-1, the transcriptional regulator of COX-2 expression, will be a potential target for the prevention of **β**-cell dysfunction mediated by PGE2.

## 1. Introduction

Cyclooxygenase-2 (COX-2) is a key enzyme that catalyzes the production of prostaglandins (PGs) and other inflammatory substances from arachidonic acid. COX-2 catalytic product PGs participate in many physiological and pathological processes, such as inflammation, pain, angiogenesis, blood pressure regulation, and immune response [1]. COX-2, as an inducible cyclooxygenase, is normally undetectable in most tissues and organs but can be rapidly induced by cytokines, growth factors, bacterial endotoxins, carcinogenic factor stimulation, and other stimuli [2–5]. The aberrant expression of COX-2 is associated with many aspects of physiological and pathological conditions such as cell malignant transformation, inflammation, cell growth and apoptosis, tumor angiogenesis, invasiveness, and metastasis [6–10]. Prostaglandin E2 (PGE2) production has been reported to impair *β*-cell function from studies in pancreatic *β*-cells and isolated islets [11–13]. Moreover, inhibition of COX-2 activity was shown to protect *β*-cell function in inflammatory factor stimulus and increased basal insulin secretion [12, 13].

In view of the important role of COX-2 in the occurrence and development of diabetes mellitus, it is necessary to progress in-depth studies on the molecular mechanisms involved in the regulation of COX-2 gene expression. At present, research about the COX-2 gene regulation mainly focused on the level of transcriptional regulation. The COX-2 promoter region contains a canonical TATA element and a number of cis-activating consensus sequences, including cAMP responsive element (CRE), E-box, NF-IL6 (CCAAT/enhancer-binding protein-*β*), AP-2, SP-1, NF-*κ*B, and STAT sites [14–21]. The specific transcription factors involved in COX-2 activation are dependent on both cell type and stimulus. For example, AP2, NF-IL-6, and CRE elements are essential for IL-1*β*-induced activation of the COX-2 gene in human microvascular endothelial cell line, HMEC-1 [15]. Moreover, NF-*κ*B transcription factor mediates the induction of COX-2 by interleukin-1 in rheumatoid synoviocytes [16]. We previously demonstrated that transcription factor Elk-1 significantly upregulated COX-2 gene promoter activity and identified several putative binding sites for Elk-1 [22].

 Elk-1 is a member of the Ets family of transcription factors. The Ets gene family conserves an 85-amino acid DNA-binding ETS domain that binds the consensus sequence 5′-GGA (A/T)-3′ in the promoter region of the target genes [23] and has various biological functions, including control of cellular proliferation, differentiation, hematopoiesis, apoptosis, tissue remodeling, angiogenesis, and transformation [24–28]. Previous studies showed that most of the Ets family members including Elk-1 are important substrates of the MAPKs, the PI3 kinases, and Ca2+ specific signaling pathways, which can be activated by growth factors or cellular stress [29]. Other studies confirmed that inducible COX-2 expression is related to the activation of the MAPKs signaling pathway [30, 31]. Thus, transcription factor Elk-1 may be an important bridge between the external stimuli and the induction of COX-2 gene expression.

 The aim of this study was to investigate the relationships between Elk-1 and COX-2. To check the relevance of our hypothesis, we first investigated the effects of Elk-1 on COX-2 transcriptional regulation and expression in the pancreatic *β*-cell line INS-1. We thus undertook to study the binding of Elk-1 to its putative binding sites in the COX-2 promoter. We also analysed glucose-stimulated insulin secretion (GSIS) in INS-1 cells that overexpressed Elk-1. 

## 2. Materials and Methods

### 2.1. Cell Line and Cell Culture

INS-1 cells were grown in RPMI 1640 medium containing 11.1 mM glucose supplemented with 10% fetal bovine serum, 10 mM HEPES, 2 mM L-glutamine, 1 mM sodium pyruvate, 50 *μ*M *β*-mercaptoethanol, 100 IU/mL penicillin, and 100 *μ*g/mL streptomycin in a humidified atmosphere (5% CO_2_, 95% air) at 37°C.

### 2.2. Plasmid Construction

The Elk-1 expression plasmid (pCMV3.0b-Elk-1) and luciferase reporter construct containing the rat COX-2 promoter (−2026/+44) were constructed in our previous study [22]. Two mutant constructs containing the sequence −2026/+44 in which nucleotides −82 to −69 were deleted or mutated from CGAGGCGGAAAGAC to CGAGGCAAGAAGAC were made by using the QuikChange II Site-Directed Mutagenesis Kit (Stratagene) according to the manufacturer's instructions. The constructs were named pCOX-2 (−2026/+44, del−82/−69) and pCOX-2 (−2026/+44, m−82/−69), respectively. All constructs were verified by DNA sequencing.

### 2.3. Cell Transient Transfection and Luciferase Assay

Transfections were performed using Lipofectamine 2000 (Invitrogen) according to the manufacturer's protocol. For luciferase assay, INS-1 cells were plated into 12-well cell culture plates 1 day before transfection. Each transfection was performed using 0.8 *μ*g luciferase reporter construct, 0.8 *μ*g Elk-1 expression plasmid or pCMV3.0b empty vector as control, and 4 ng Renilla luciferase reporter vector, pRL-SV40, as an internal control (Promega). 48 h after transfection, cells were washed with PBS and lysed using 1× passive lysis buffer. Firefly and Renilla luciferase activities were measured with a GloMax-20/20 luminometer (Promega) using the Dual-Luciferase Reporter Assay System (Promega). Firefly luciferase activity was normalized to Renilla luciferase activity. Each experiment was performed in triplicate and independently repeated three times.

 To explore the effect of Elk-1 on endogenous COX-2 expression and glucose-stimulated insulin secretion of INS-1 cells, cells were transiently transfected with Elk-1 expression plasmid pCMV3.0b- Elk-1 or empty vector pCMV3.0b as control. 48 h after transfection, the cells were harvested for quantitative real-time RT-PCR or Western blot analysis, or proceed to glucose-stimulated insulin secretion (GSIS) assay as follows.

### 2.4. Quantitative Real-Time RT-PCR

Total RNAs of INS-1 cells were prepared by TRIzol reagent (Invitrogen) according to the manufacturer's protocol. After spectrophotometry quantification, 1 *μ*g of total RNA was used for reverse transcription (RT) in a 20 *μ*L final volume with iScript cDNA Synthesis Kit (Bio-Rad) according to the manufacturer's instructions. Quantitative real-time PCR was performed using TaqMan Gene Expression Assays (Applied Biosystems) in a StepOnePlus Real-Time PCR System (Applied Biosystems). The reactions were performed in a volume of 10 *μ*L containing 1 *μ*L diluted cDNA, 20× TaqMan Gene Expression Assay Mix, and 2× TaqMan Universal PCR Master Mix. The thermal cycling conditions comprised an initial denaturation step at 95°C for 10 min, 40 cycles at 95°C for 15 s, and 60°C for 1 min. The TaqMan Gene Expression Assay Mix used for Elk-1 and COX-2 had the product number Rn01756649_g1 and Rn01483828_m1. Rat *β*-actin (product number Rn00667869_m1) was used to calibrate the original concentration of mRNA. Each quantification PCR was performed in triplicate and independently repeated three times. The mRNA concentration was defined as the ratio of target mRNA copies relative to GAPDH mRNA copies.

### 2.5. Western Blot Analysis

INS-1 cells were lysed in ice-cold lysis buffer containing the following reagents: 50 mM Tris-HCl pH 7.4; 1% NP-40; 150 mM NaCl; 1 mM EDTA; 1 mM PMSF; complete proteinase inhibitor mixture (1 tablet per 10 mL, Roche). Protein concentration in the cell lysate was quantified using the DC Protein Assay Kit (Bio-Rad). Protein aliquots were electrophoresed by 12% SDS-PAGE and transferred to PVDF membrane (Millipore). Nonspecific protein interactions were blocked by incubation in 5% nonfat dry milk in TBST buffer (20 mM Tris-HCl, 150 mM NaCl, and 0.1% Tween 20 (pH 7.6)) at room temperature for 1 h and then washed with TBST. Membranes were then incubated at 4°C overnight with anti-Elk-1 (Santa Cruz), anti-COX-2 (Santa Cruz), or anti-*β*-actin (Santa Cruz) antibodies in fresh blocking buffer. The blots were washed and then incubated with HRP-conjugated secondary antibodies (Amersham) for 1 h at room temperature. The bands were visualized with Immobilon Western Chemiluminescent HRP Substrate (Millipore) using X-ray film (Kodak). Prestained markers (Thermo) were used as internal molecular weight standards. The densities of the bands on the Western blot were analyzed with Quantity One software (Bio-Rad). 

### 2.6. Knockdown of Elk-1 by RNA Interference (RNAi)

Elk-1 specific small interfering RNA (siRNA) and negative siRNA were synthesized by GenePharma. The sequences were as follows: Elk-1 siRNA-1, 5′-GGCCAGAAGUUUGUCUACAtt-3′; siRNA-2, 5′-AGGCCAAGGUGGCUUAGCAtt-3′; siRNA-3, 5′-GCCAUCCUAACAGAGAAUAtt-3′; negative siRNA, 5′-UUCUCCGAACGUGUCACGUtt-3′. INS-1 cells were transiently transfected with siRNA using NeoFx reagent (Ambion) according to the manufacturer's protocol. 48 h after transfection, the cells were harvested for real-time RT-PCR or Western blot analysis as described above.

### 2.7. Nuclear Protein Extraction and Electrophoretic Mobility Shift Assay (EMSA)

Nuclear extracts were isolated from INS-1 cells with NE-PER nuclear and cytoplasmic extraction reagents (Pierce) according to the manufacturer's instructions. Protein concentration was determined with DC Protein Assay Kit (Bio-Rad). EMSA was performed using DIG Gel Shift Kit (Roche) according to the manufacture's protocol. The sense probe sequences for EMSA were as follows: wild-type probe 1, 5′-AAAGCCGAGGCGGAAAGACACAGT-3′, which corresponds to nucleotide −87 to −64 of rat COX-2 promoter; wild-type probe 2, 5′-TTCGGTAGTTTCCGAAGGGCTGTT-3′, which corresponds to nucleotide −1300 to −1277 of COX-2 promoter; wild-type probe 3, 5′-ACCACCCATTTCCGACCCCCCACC-3′, which corresponds to nucleotide −1824 to −1801 of COX-2 promoter; mutant probe 1, 5′-AAAGCCGAGGCAAGAAGACACAGT-3′. Double-stranded probes were synthesized, and the 3′-end of wild-type probe was labelled with digoxigenin-11-ddUTP. Nuclear extracts (5 *μ*g protein) were incubated with 1 *μ*g poly[d (I-C)], the binding buffer attached to the kit, and DIG-labelled wild-type probe for 15 min at room temperature. Bound DNA complexes were separated on a 5% nondenaturing polyacrylamide gel electrophoresis and transferred to a nylon membrane (Roche). The nylon membranes were cross-linked, and chemiluminescent detection was performed using CSPD, and signals were recorded on X-ray film.

 In supershift analyses, Elk-1 antibody (3 *μ*g; Santa Cruz) was added to nuclear extracts in gel shift buffer (above) for 1 h at 4°C, followed by addition of probe, and the subsequent protocol was the same as above.

### 2.8. GSIS Assay

One day before transfection, INS-1 cells (2 × 10^5^) were seeded into 500 *μ*L RPMI 1640 medium with standard glucose concentration (11.1 mM) in 24-well cell culture plates. The cells were transfected with Elk-1 expression plasmid pCMV3.0b- Elk-1 or empty vector pCMV3.0b as control for 48 h as described above. After incubation for 1 h in glucose-free Krebs-Ringer bicarbonate (KRB) buffer (115 mM NaCl, 4.7 mM KCl, 1.2 mM MgSO_4_·7H_2_O, 1.2 mM KH_2_PO_4_, 20 mM NaHCO_3_, 16 mM HEPES, 2.56 mM CaCl_2_, and 0.2% BSA), the cells were treated for 1 h in KRB buffer with low (3.3 mM) and high (16.7 mM) glucose. The supernatants were obtained for insulin concentration determination using a rat/mouse insulin ELISA kit (Linco Research). Each experiment was performed in triplicate and independently repeated three times.

### 2.9. Statistical Analysis

Data were presented as mean ± SEM. Differences in the mean of two samples were analysed by Student's *t*-test with differences *P* < 0.05 considered significant. Statistical analysis was performed with SPSS 17.0 software.

## 3. Results

### 3.1. Elk-1 Upregulated COX-2 Gene Expression

**Figure 1:**
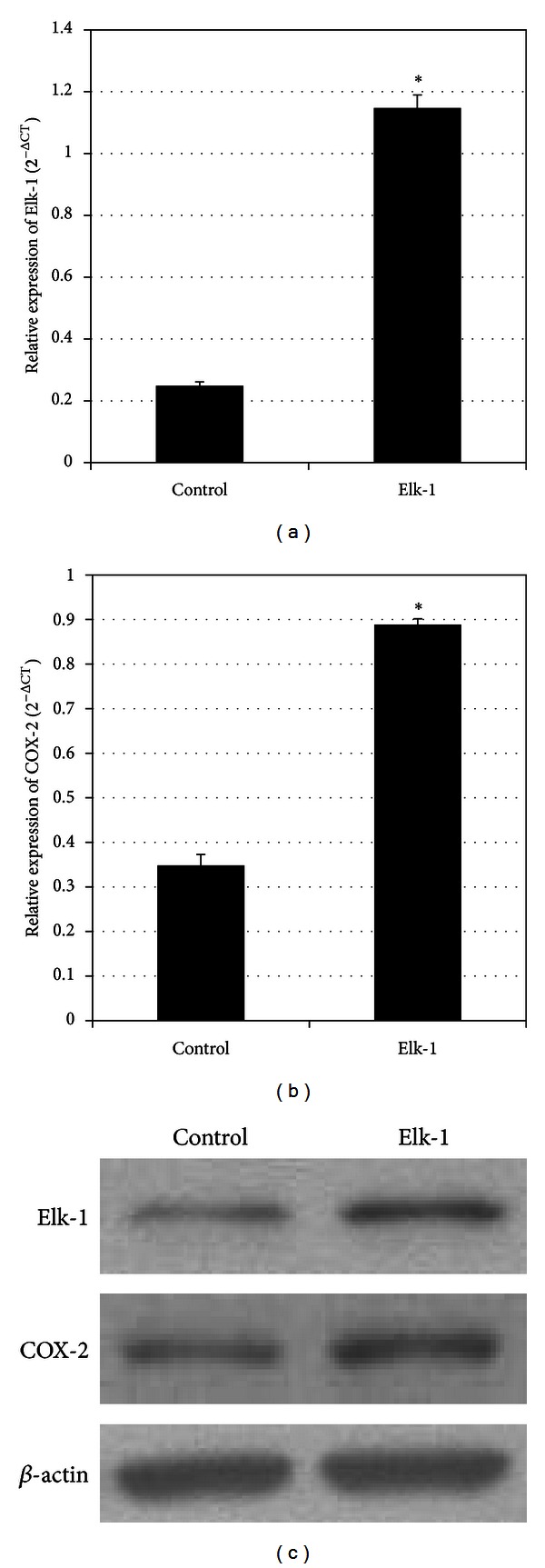
Elk-1 upregulated COX-2 gene expression. (a and b) Elk-1 and COX-2 mRNA levels were determined by quantitative real-time RT-PCR. Relative mRNA expression was expressed as mean ± SEM. *P < 0.001 versus control. (c) Elk-1 and COX-2 protein levels were assayed by Western blot analysis. *β*-actin levels served as internal control.

In the previous study, we demonstrated that transcription factor Elk-1 significantly upregulated COX-2 gene promoter activity [22]. To explore the effect of Elk-1 on endogenous COX-2 gene expression, INS-1 cells were transiently transfected with Elk-1 overexpression vector or control vector. Overexpression of Elk-1 significantly increased COX-2 mRNA and protein expression (Figure 1).

### 3.2. Elk-1 RNAi Downregulated COX-2 Gene Expression

**Figure 2:**
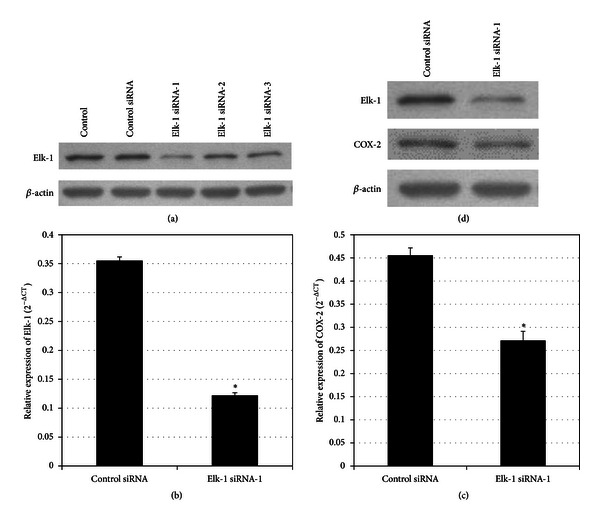
Elk-1 RNAi downregulated COX-2 gene expression. (a) INS-1 cells were transiently transfected with control siRNA and Elk-1 siRNAs (1, 2, and 3), respectively. Untransfected cells were used as control. 48 h after transfection, Elk-1 protein levels were assayed by Western blot analysis. *β*-actin levels served as internal control. (b and c) INS-1 cells were transiently transfected with control siRNA and Elk-1 siRNA-1, respectively. 48 h after transfection, Elk-1 and COX-2 mRNA levels were determined by quantitative real-time RT-PCR. Relative mRNA expression was expressed as mean ± SEM. *P < 0.001 versus control siRNA transfected group. (d) Elk-1 and COX-2 protein levels were assayed by Western blot analysis. *β*-actin levels served as internal control.

INS-1 cells were transfected either with Elk-1 siRNAs (including 3 siRNAs) or control siRNA. We measured Elk-1 protein levels to select the siRNAs that can effectively silence Elk-1 expression. As shown in Figure 2(a), Elk-1 siRNA-1 effectively silenced Elk-1 gene expression; therefore, we used Elk-1 siRNA-1 in subsequent experiments. We silenced Elk-1 expression in INS-1 cells and then measured COX-2 mRNA and protein expression. COX-2 mRNA and protein expression levels were significantly decreased with Elk-1 RNAi (Figures 2(b), 2(c), and 2(d)).

### 3.3. Identification of Elk-1 Binding Site in COX-2 Promoter

**Figure 3:**
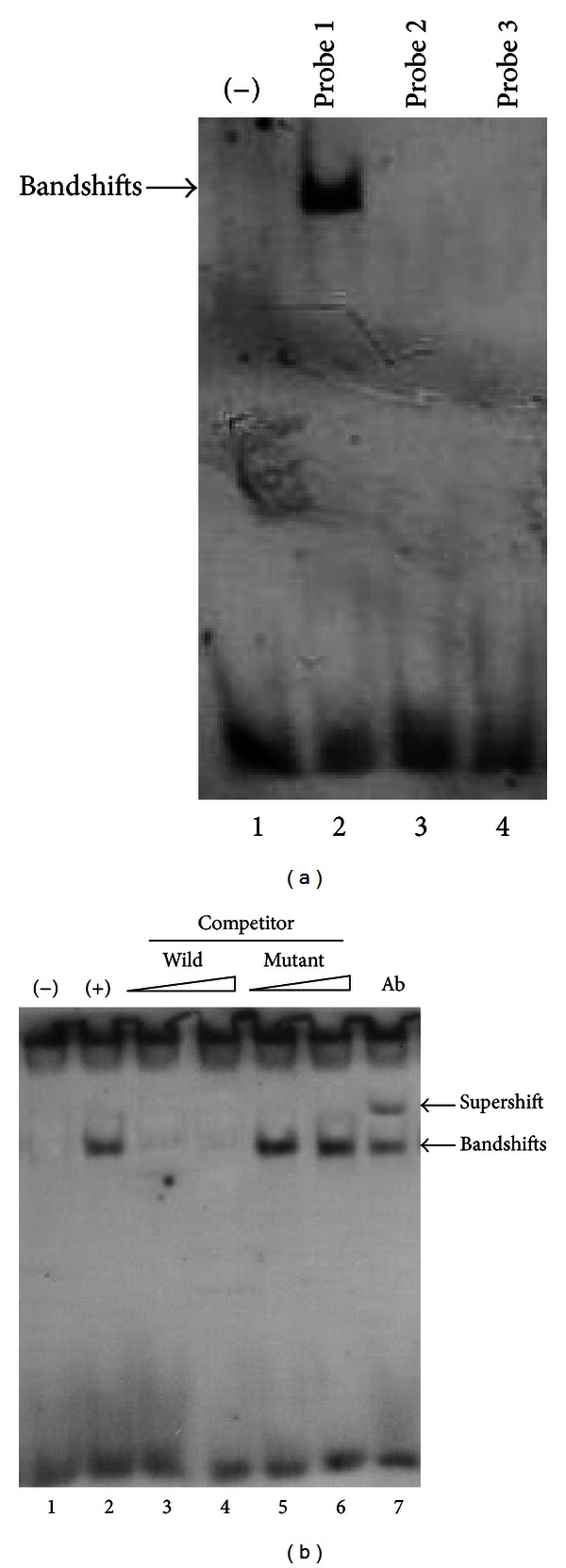
Identification of Elk-1 binding site in COX-2 promoter by electrophoretic mobility shift assay (EMSA). (a) Wild-type probe 1 (−87/−64 of COX-2 promoter) was incubated without (lane 1) or with (lane 2) INS-1 nuclear proteins. Wild-type probe 2 (−1300/−1277) (lane 3) or wild-type probe 3 (−1824/−1801) (lane 4) was incubated with INS-1 nuclear proteins. (b) Wild-type probe 1 was incubated without (lane 1) or with (lane 2) INS-1 nuclear proteins in the absence or presence of unlabelled probe 1 (lanes 3–6). Lanes 3 and 4 contain the wild-type probe 1, and lanes 5 and 6 contain the mutant probe 1, each at 50- and 100-fold molar excess, respectively. In lane 7, wild-type probe 1 was incubated with INS-1 nuclear proteins in the presence of Elk-1 antibody.

Based on sequence analysis, rat COX-2 promoter region contains three predicted consensus binding sites for Elk-1, which correspond to promoter region of −82/−69, −1295/−1282, and −1819/−1806, respectively. To confirm whether Elk-1 can bind to these three sites, we synthesized and labelled the oligonucleotides spanning the three regions and additional five nucleotides on each side (i.e., −87/−64, −1300/−1277, and −1824/−1801) and used them as probes in EMSA experiments. As shown in Figure 3(a) (lane 2), a slower-migrating complex appeared when INS-1 nuclear extracts were incubated with the digoxigenin-11-ddUTP-labelled wild-type probe 1 (−87/−64 of COX-2 promoter), but not probe 2 and probe 3 (−1300/−1277 and −1824/−1801), indicating that −82/−69 region is the binding site for Elk-1. The slower-migrating complex was significantly inhibited by a molar excess of unlabelled wild-type probe 1 (Figure 3(b), lanes 3 and 4). In contrast, the unlabelled mutant probe 1 reduced the inhibitory effect (Figure 3(b), lanes 5 and 6). To determine if Elk-1 is responsible for the shift seen in EMSA, Elk-1 antibody was added to the EMSA-binding reaction, and the complex can be supershifted by the addition of Elk-1 antibody (Figure 3(b), lane 7).

### 3.4. Elk-1 Upregulated COX-2 Promoter Activity through Elk-1 Binding Site

**Figure 4:**
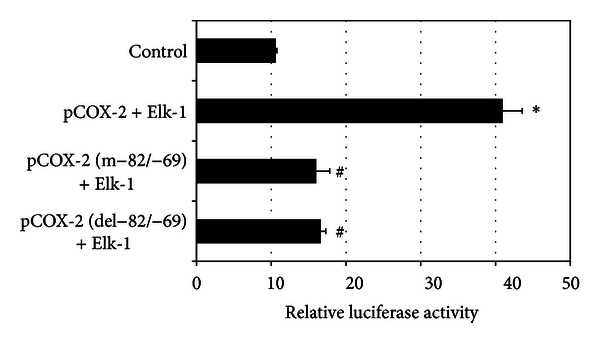
Elk-1 upregulated COX-2 promoter activity through Elk-1 binding site. The Elk-1 expression vector (pCMV3.0b-Elk-1) or control vector (pCMV3.0b) was transfected together with construct pCOX-2 (−2026/+44), pCOX-2 (−2026/+44, m−82/−69), or pCOX-2 (−2026/+44, del−82/−69). Relative luciferase activity was expressed as mean ± SEM and represented three different experiments in triplicate for each fragment. *P < 0.001 versus control. ^#^ *P* < 0.001 versus pCOX-2 (−2026/+44) transfacted with pCMV3.0b- Elk-1.

By transient cotransfections, we showed that overexpression of Elk-1 led to a significant increase in relative luciferase activity of COX-2 promoter (Figure 4). The contribution of the Elk-1 binding site was studied by site-directed mutagenesis in INS-1 cells. When the −82/−69 Elk-1 binding site on the −2026/+44 region was deleted or mutated, the enhanced effect of Elk-1 was deeply reduced (Figure 4). This suggested that the −82/−69 Elk-1 binding site is implicated in the enhancement of COX-2 promoter by Elk-1.

### 3.5. Elk-1 Inhibited Glucose-Stimulated Insulin Secretion in INS-1 Cells

**Figure 5:**
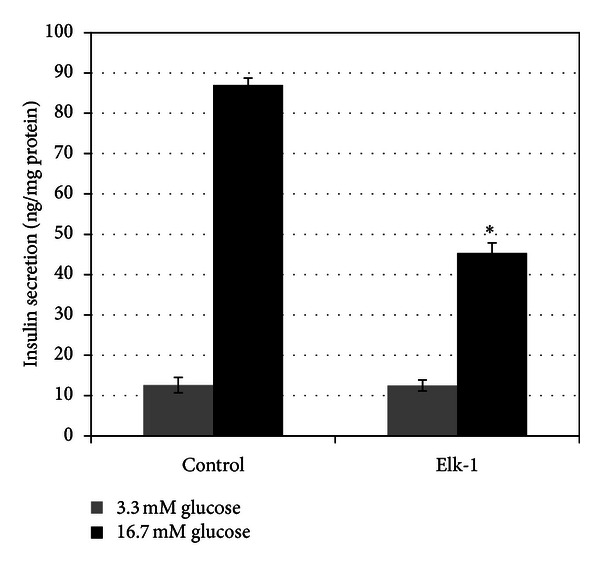
Elk-1 inhibited glucose-stimulated insulin secretion in INS-1 cells. INS-1 cells were transfected with Elk-1 expression plasmid pCMV3.0b-Elk-1 or empty vector pCMV3.0b as control for 48 h. Control cells demonstrated a 6.9-fold increase in insulin secretion with 16.7 mM glucose, whereas Elk-1 overexpressing cells had only a 3.6-fold increase. Elk-1 overexpressing group demonstrated a decrease of GSIS to 52% of the control value. Each experiment was done in triplicate and repeated three times. *P < 0.001 versus control.

To determine the effect of Elk-1 on GSIS function in pancreatic *β*-cells, we performed experiments with Elk-1 overexpressing INS-1 cells. As shown in Figure 5, control cells secreted 87.00 ± 1.74 ng insuli·nh^−1^·mg protein^−1^ and demonstrated a 6.9-fold increase in insulin secretion with 16.7 mM glucose, whereas Elk-1 overexpressing cells secreted 45.37 ± 2.50 ng insulin·h^−1^·mg protein^−1^ and demonstrated a 3.6-fold increase in insulin secretion (*P* < 0.001 versus control). Therefore, Elk-1 overexpressing group demonstrated a decrease of GSIS to 52% of the control value.

## 4. Discussion

COX-2 is an immediate early gene. Depending on the cell type, it can be activated by a variety of stimuli. Upregulation of COX-2 expression is involved in various physiological and pathological conditions, including pancreatic *β*-cell dysfunction. Previous studies indicated that COX-2 activation might play a pathogenic role in diabetes [32–34], and COX-2 inhibition can protect rat islets from cytokine-induced inhibition of glucose-stimulated insulin secretion [13], implicating the important role of COX-2 in cytokine-mediated *β*-cell dysfunction and diabetes development. Thus, understanding the molecular mechanisms involved in the regulation of COX-2 gene expression in *β*-cells will help to better understand and restrain the dysfunction of pancreatic *β*-cell.

 COX-2 expression is regulated by the binding of specific transcription factors to cis-acting elements on the COX-2 promoter [35]. Several studies showed that some stimuli could upregulate COX-2 expression, and in these studies, the expression and activity of Elk-1 were also increased [36–38], indicating the potential role of Elk-1 in COX-2 regulation. In our previous study, we demonstrated that Elk-1 significantly upregulated COX-2 promoter activity [22]. But how Elk-1 participates in the regulation of COX-2 expression has not been researched so far.

In this study, we investigated the effects of Elk-1 on COX-2 gene expression and GSIS function in INS-1 rat insulinoma cells and explored whether Elk-1 regulates COX-2 expression through its potential binding site in COX-2 promoter. Overexpression study demonstrated that Elk-1 overexpression significantly increased COX-2 mRNA and protein expression. On the contrary, Elk-1 RNAi significantly decreased COX-2 expression. EMSA and site-directed mutagenesis experiments indicated that the effect of Elk-1 on COX-2 transcription involves one Elk-1 cis-element of COX-2 promoter located between nucleotides −82 and −69, but not the other two predicted consensus cis-elements (−1295/−1282 and −1819/−1806). These results suggested that Elk-1 probably upregulates COX-2 gene expression at least partly through Elk-1 directly binding to the −82/−69 region of COX-2 promoter. This was the first report that characterized the Elk-1 cis-element in COX-2 promoter.

To further investigate the role of Elk-1 on pancreatic *β*-cells dysfunction, we assessed GSIS function in INS-1 cells that overexpressed Elk-1. As expected, Elk-1 overexpressing group demonstrated a decrease of GSIS to 52% of the control value. This result demonstrated that Elk-1 can inhibit GSIS in INS-1 cells. Because COX-2 plays an important role in *β*-cell dysfunction and Elk-1 can upregulate COX-2 gene expression, we presumed that Elk-1 inhibits GSIS in INS-1 cells at least partly through upregulating COX-2 gene expression. Other unknown mechanisms of Elk-1's inhibitory role on GSIS remain to be determined by further experiments such as chromatin immunoprecipitation (ChIP) sequencing, transcriptome sequencing, and gene array.

## 5. Conclusions

In conclusion, our study demonstrated that transcription factor Elk-1 efficiently upregulates the expression of COX-2 in INS-1 cells and that this may be a new explanation for the mechanism of *β*-cell insulin secretion impairments by some stimuli. Overexpression of Elk-1 may inhibit insulin secretion in *β*-cells by causing upregulation of COX-2. These findings will be helpful for better understanding the transcriptional regulation of COX-2 in pancreatic *β*-cell. Moreover, Elk-1, the transcriptional regulator of COX-2 expression, will be a potential target for the prevention of *β*-cell dysfunction mediated by PGE2.
